# Schiff Bases Functionalized with T-Butyl Groups as Adequate Ligands to Extended Assembly of Cu(II) Helicates

**DOI:** 10.3390/ijms24108654

**Published:** 2023-05-12

**Authors:** Sandra Fernández-Fariña, Isabel Velo-Heleno, Miguel Martínez-Calvo, Marcelino Maneiro, Rosa Pedrido, Ana M. González-Noya

**Affiliations:** 1 Departamento de Química Inorgánica, Facultade de Química, Campus Vida, Universidade de Santiago de Compostela, 15782 Santiago de Compostela, Spain; mariaisabel.velo.heleno@usc.es (I.V.-H.); miguel.martinez.calvo@usc.es (M.M.-C.); ana.gonzalez.noya@usc.es (A.M.G.-N.); 2 Departamento de Química Inorgánica, Facultade de Ciencias, Universidade de Santiago de Compostela, 27002 Lugo, Spain; marcelino.maneiro@usc.es

**Keywords:** Schiff bases, copper, helicates, magnetic properties

## Abstract

The study of the inherent factors that influence the isolation of one type of metallosupramolecular architecture over another is one of the main objectives in the field of Metallosupramolecular Chemistry. In this work, we report two new neutral copper(II) helicates, [Cu_2_(L^1^)_2_]·4CH_3_CN and [Cu_2_(L^2^)_2_]·CH_3_CN, obtained by means of an electrochemical methodology and derived from two Schiff-based strands functionalized with ortho and para-t-butyl groups on the aromatic surface. These small modifications let us explore the relationship between the ligand design and the structure of the extended metallosupramolecular architecture. The magnetic properties of the Cu(II) helicates were explored by Electron Paramagnetic Resonance (EPR) spectroscopy and Direct Current (DC) magnetic susceptibility measurements.

## 1. Introduction

The search for new routes to obtain new metallosupramolecular architectures and the study of their potential applications is a field of great interest in Metallosupramolecular Chemistry. The knowledge of the different factors that influence the self-assembly process is essential to control the obtainment of a specific type of compound, so it is necessary to deepen our understanding by designing new systems. Among all factors, the ligand design directly influences the structure of the final metallosupramolecular architecture and thus its properties and applications [1].

The term “helicate” was introduced by Jean-Marie Lehn in 1987 to describe a class of copper(I) compounds exhibiting a helicoidal architecture with similar characteristics to the DNA double helix [2]. A helicate consists of one or more organic ligands that wrap helically around a series of metal ions that define the helix axis [3].

To obtain helicoidal architectures, the precursor ligands should contain two or more binding domains separated by a flexible spacer to allow helical coiling, but also it should be rigid enough to prevent multiple binding domains coordinating to the same metal ion, giving rise to mononuclear species [4,5,6]. Moreover, it was demonstrated that the isolation of helicoidal architectures over other possible arrangements can be controlled by the intra- and intermolecular interactions established by the ligand units [7,8]. In the literature there is a large variety of examples of helicate-type extended architectures whose formation is favored and determined by the existence of weak non-covalent π-π or CH···π interactions [4,9].

Currently, the research on metal helicates is mainly directed towards the search of their potential applications [10,11,12,13]. Among these properties there is special interest on those helicates exhibiting relevant magnetic behaviors that could be used as new magnetic materials [14,15,16]. However, the factors that selectively lead to a particular type of metallosupramolecular compound, and to helicates in particular, continue to be of interest and deserve to be further investigated. 

Schiff base ligands have been extensively used in Coordination Chemistry [17,18,19] and more particularly in Metallosupramolecular Chemistry to obtain helicates [9,11], with some of them showing relevant biomedical [20] or photophysical [11] properties. In this context, these types of ligands were employed by our research group to obtain the first example of a network assembled from Cu(II) helicates through intermolecular π-π interactions showing antiferromagnetic behavior [21]. In this primary work the antiferromagnetic character was attributed to the establishment of weak π-π interactions between neighboring helicate units. 

With this precedent in mind, in an attempt to explore the relationship between the ligand design, the extended helical structure and the magnetic properties, we approach the obtainment of helicates combining Schiff base ligands, copper(II) ions and an electrochemical methodology. Herein, we report two novel copper(II) helicates derived from two Schiff base ligands substituted with t-butyl groups and their crystal structures. We studied their magnetic properties by EPR spectroscopy and DC magnetic susceptibility.

## 2. Results and Discussion

### 2.1. Synthesis and Characterization of the Ligands H_2_L^1^ and H_2_L^2^

**Scheme 1:**
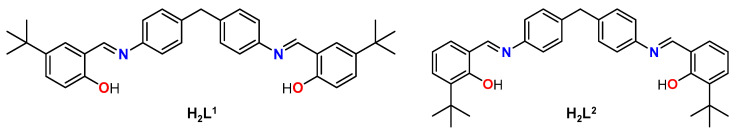
Schiff base ligands H_2_L^1^ and H_2_L^2^.

In the present work, we approach the obtainment of extended helicates using dianiline-derived Schiff base ligands. It should be highlighted that the long and semi-flexible dianiline-type spacers have been widely used by Hannon and co-workers, proving to be an effective unit for obtaining a wide variety of helicoidal architectures using different metal ions [22,23,24]. For this purpose, we designed two new Schiff base ligands containing the dianiline spacer and two terminal hydroxybenzaldehyde rings decorated with ortho and para-tert-butyl groups (H_2_L^1^ and H_2_L^2^, Scheme 1).

The ligands H_2_L^1^ and H_2_L^2^ are potentially dianionic with two bidentate [NO] domains separated by a semi-flexible aromatic spacer, factors that should favor the isolation of helical-type complexes. The main objective is to find out whether the position of the tert-butyl group influences the final discrete and extended architecture and the magnetic properties of the final compounds. 

**Scheme 2:**
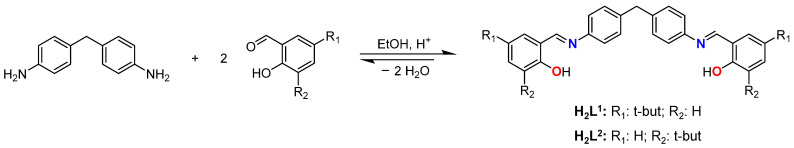
Synthesis of the Schiff base ligands H_2_L^1^ and H_2_L^2^.

Both ligands were synthesized by a reaction between the corresponding hydroxybenzaldehyde functionalized with tert-butyl groups [25] and 4,4′-methylenedianiline in a 2:1 ratio, using absolute ethanol as a solvent (Scheme 2). H_2_L^1^ and H_2_L^2^ were fully characterized by melting point determination, elemental analysis, infrared spectroscopy, mass spectrometry and ^1^H NMR spectroscopy techniques (Figures S1 and S2, Supplementary Material).

### 2.2. Synthesis and Characterization of the Copper Complexes

Two neutral copper complexes were isolated from H_2_L^1^ and H_2_L^2^ using an electrochemical methodology (see details in experimental section and in references [26,27,28]). The electrochemical synthesis of the neutral metal complexes was carried out by oxidation of a copper plate in a conductive solution of the corresponding ligand in acetonitrile. The efficiency values calculated for the electrochemical synthesis of both complexes have values around 0.5 mol·F^−1^, so the proposed mechanism would involve the loss of two electrons for each metal atom, as shown below:

Cathode: 2 H_2_L^1|2^ + 4 e^−^ → 2 (L^1|2^)^2−^ + 2 H_2_(g) Anode: 2 Cu → 2 Cu^2+^ + 4 e^−^ Global: 2 (L^1|2^)^2−^ + 2 Cu^2+^ → Cu_2_(L^1|2^)_2_

The resulting brown solid complexes were characterized by melting point determination, elemental analysis, infrared spectroscopy, X-ray diffraction and mass spectrometry (Figures S3–S6, Supplementary Material).

Both analytical and spectroscopic data allow us to propose dinuclear stoichiometries of the type [Cu_2_(L^1|2^)_2_], with the ligands being coordinated to the copper centers in their dianionic form [L^1|2^]^2−^. The infrared spectra of both complexes exhibit a slight shift of the characteristic bands of the ligand skeletons to lower wavenumbers due to the coordination of the metal ions. More in detail, a variation in the ν(C=N) band is observed, indicating that the ligand is bound to the metal via the imine nitrogen atoms. In addition, the increase in intensity and the shift of the vibration band (C-O) suggest the coordination of the copper(II) ions through the phenolic oxygen atoms of the ligand. Similarly, it is observed that both vibrational bands, ν(C-H_Ar_) and ν(CH_2_), increase in intensity due to the effect of the coordination. The formation of the copper(II) complexes derived from the Schiff base ligands H_2_L^1^ and H_2_L^2^ was also confirmed by MALDI-TOF (+) mass spectrometry (Figure S4), as the peaks corresponding to the dinuclear fragments [Cu_2_L_2_ + H]^+^ are observed in the mass spectra of both complexes.

#### X-ray Structures

**Figure 1:**
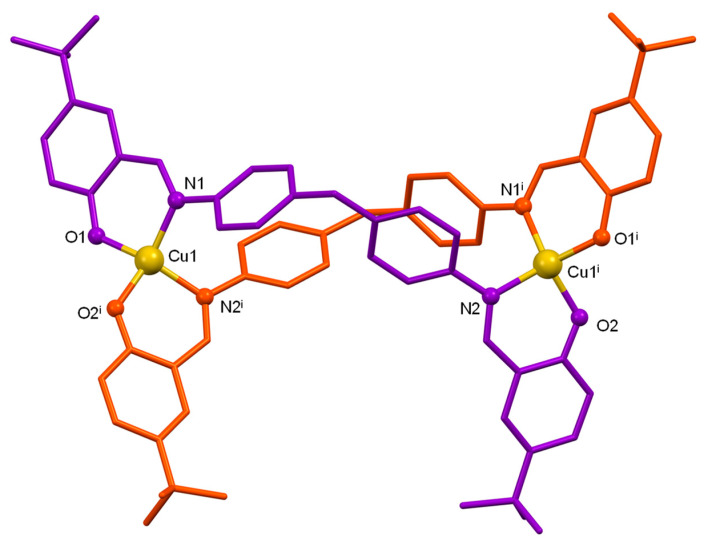
Crystal structure of the copper(II) helicate [Cu_2_(L^1^)_2_]·4CH_3_CN. Solvent molecules and hydrogen atoms were omitted for clarity.

**Figure 2:**
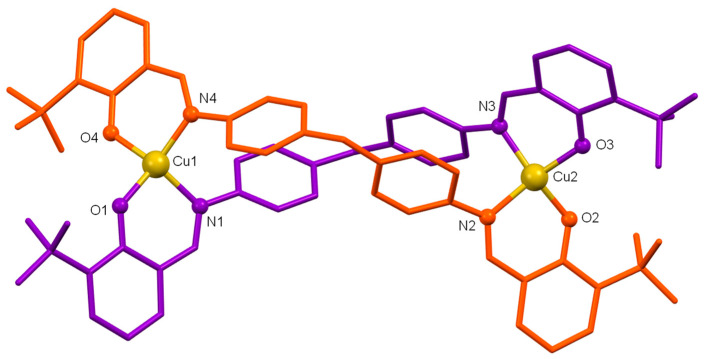
Crystal structure of the copper(II) helicate [Cu_2_(L^2^)_2_]·CH_3_CN. Solvent molecules and hydrogen atoms were omitted for clarity.

Slow evaporation of the mother liquors from the synthesis of [Cu_2_(L^1^)_2_] and [Cu_2_(L^2^)_2_]·CH_3_CN complexes allowed us to achieve good-quality crystals for X-ray diffraction studies. The crystal structures of the complexes ([Cu_2_(L^1^)_2_]·4CH_3_CN and [Cu_2_(L^2^)_2_]·CH_3_CN are depicted in Figure 1 and Figure 2. Table S1 contains the main crystallographic data for these complexes, whereas Tables S2–S5 summarizes the most relevant distances and angles.

The discrete crystal structures of both compounds are similar, so a joint discussion is performed here, highlighting differences. Both structures show neutral dinuclear helicate-type architectures formed by two strands of the bideprotonated ligand [L^1|2^]^2−^ that cross each other when coordinating the two Cu(II) ions (Figure 1 and Figure 2). The ligands act in such a way that each of their bidentate [NO] branches coordinate to a different metal ion, giving rise to a distorted tetrahedral geometry (≠109.5°) for the Cu(II) ions. The O-M-N bond angles clearly show the distortion of the tetrahedral geometry (Tables S2 and S3).

The main bond distances Cu-O and Cu-N are in the expected ranges for Cu(II) complexes derived from Schiff base ligands with phenol groups [29], with the bond distance of Cu-O being slightly smaller than Cu-N (see Tables S2 and S3). The intermetallic distances of Cu···Cu (11.76 Å for [Cu_2_(L^1^)_2_]·4CH_3_CN and 11.87 Å for [Cu_2_(L^2^)_2_]·CH_3_CN) are in the order of those found for other Cu(II) helicates with dianiline-type spacers and do not deserve further comments [30].

**Figure 3:**
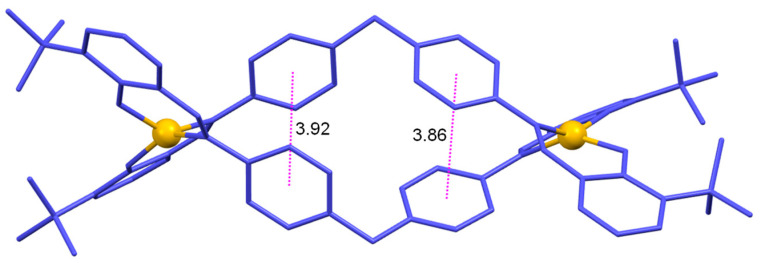
The π-π interactions between the aromatic rings of the two ligands in the copper(II) helicate [Cu_2_(L^2^)_2_]·CH_3_CN.

Each helicate molecule displays eight aromatic rings, which makes possible the establishment of aromatic π-π or CH···π stacking interactions. Thus, both copper(II) helicates display weak π-π interactions between the aromatic rings of the two aniline spacers that contribute to the stabilization of the helicoidal structure (distance between centroids: 3.890 Å for [Cu_2_(L^1^)_2_]·4CH_3_CN; 3.92 Å and 3.86 Å for [Cu_2_(L^2^)_2_]·CH_3_CN, Figure 3). 

**Figure 4:**
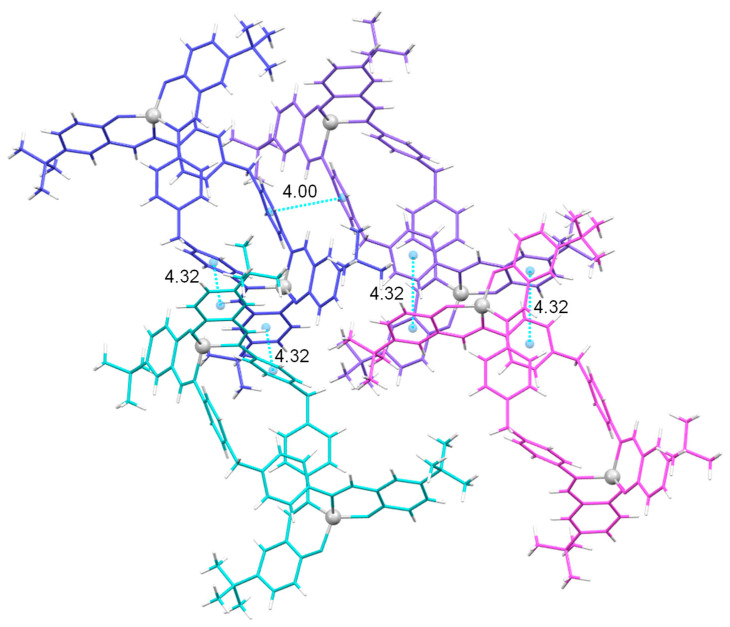
Intermolecular π-stacking interactions in the crystal lattice of the complex [Cu_2_(L^1^)_2_]·4CH_3_CN.

The crystal lattice of [Cu_2_(L^1^)_2_]·4CH_3_CN (Figure 4) shows intermolecular π-π interactions involving the aromatic rings of the spacer of adjacent helicate units (centroid–centroid distance 4.00 Å), with these interactions being similar to the intramolecular ones (3.89 Å). These interactions are also observed between one of the phenyl rings of the spacer and the aromatic ring of a linker domain (centroid–centroid distance 4.32 Å (Figure 4). In addition, CH···π interactions between the aromatic ring of one of the ligand branches and one of the tert-butyl substituents of the adjacent helicate can be observed in the [Cu_2_(L^1^)_2_]·4CH_3_CN (3.79 Å) cell (Figure S4, Supplementary Material).

It should be noted that the only interaction that can be observed in the crystal lattice of the helicate [Cu_2_(L^2^)_2_]·CH_3_CN involves one of the phenyl rings of the spacer, with the benzene of a linker domain (centroid–centroid distance 3.79 Å) being an important difference compared to the [Cu_2_(L^1^)_2_]·4CH_3_CN helicate (Figure S5).

**Figure 5:**
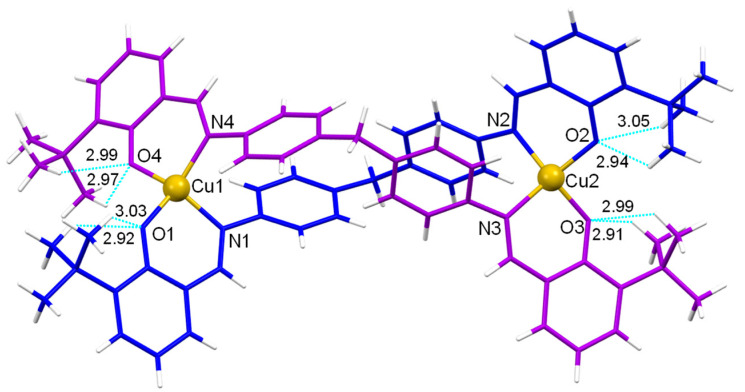
Crystal structure of the [Cu_2_(L^2^)_2_]·CH_3_CN helicate showing the intramolecular hydrogen bonds: C29−H29B···O1 2.92 Å, C30−H30C···O1 3.03 Å, C69−H69B···O4 2.97 Å, C70−H70C···O4 2.99 Å, C34−H34C···O2 2.94 Å, C33−H33B···O2 3.05 Å, C64−H64B···O3 2.91 Å and C65−H65C···O3 2.99 Å.

In addition, the copper(II) helicate [Cu_2_(L^2^)_2_]·CH_3_CN, which incorporates the tert-butyl groups adjacent to the phenolic groups, establishes hydrogen bond interactions between the CH_3_ of the tert-butyl groups and the phenolic oxygen atoms (Figure 5) [31].

It is remarkable to mention that in the case of the two helicates described in this work the distance between the Cu(II) ions of the closest stacked helicates (intermolecular metal distance) is notably smaller than the distance between the two metal atoms within the molecule, in the same way as the copper(II) helicate reported by us in 2003 [21] and the cobalt(II) helicate reported later on by Andruh and co-workers [32]. This interesting structural arrangement could affect the magnetic properties of the two helicates, as discussed below.

**Figure 6:**
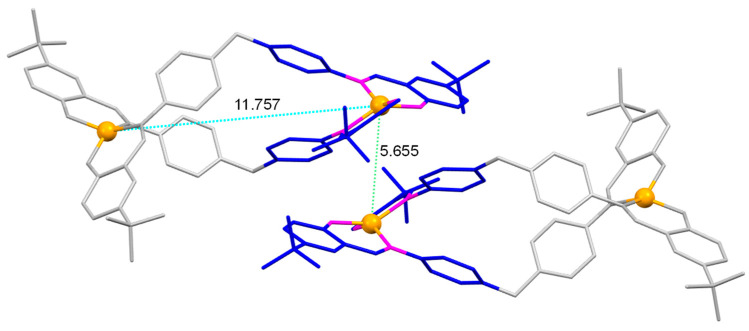
Part of the crystalline cell of the [Cu_2_(L^1^)_2_]·4CH_3_CN helicate, showing the π-π type interactions (rings in blue), as well as the intramolecular (blue) and intermolecular M-M distances with the metal ions of the adjacent ligand (green).

**Figure 7:**
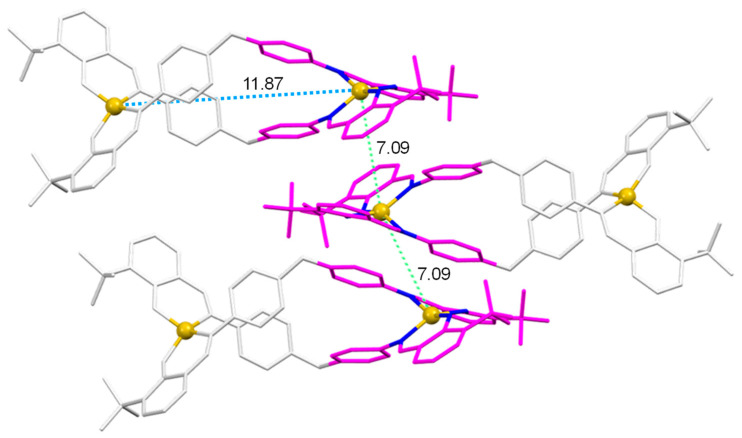
Part of the crystalline cell of the [Cu_2_(L^2^)_2_]·CH_3_CN helicate, showing the π-π type interactions (pink rings), as well as the intramolecular (blue) and intermolecular M-M distances with the metal ions of the closest helicates (green).

It is also worth mentioning that the intermolecular distance between metal ions is smaller in the case of the [Cu_2_(L^1^)_2_]·4CH_3_CN helicate (~5.6 Å) (Figure 6), which exhibits the tert-butyl substituent in the para position with respect to the phenolic oxygen, compared with that in the [Cu_2_(L^2^)_2_]·CH_3_CN helicate, which incorporates the tert-butyl substituent in the ortho position (~7.1 Å) (Figure 7).

All this information confirms that the highly aromatic Schiff base ligands H_2_L^1^ and H_2_L^2^ are suitable to obtain extended helicate structures through weak intermolecular interactions. In addition, the position of the bulky t-butyl groups influences the microarchitecture of the extended structure, as demonstrated by the shorter intermolecular Cu---Cu distance displayed when the ligand exhibits the tert-butyl groups far away from the binding sites (para position).

### 2.3. Magnetic Properties of Helicates

It is well known that in coordination compounds metal ions can interact with each other when the distance between them is small [33]. Additionally, in the literature there are examples of helicoidal supramolecular architectures with large intramolecular M-M distances showing relevant magnetic behavior, for which interesting nanotechnological applications are proposed. As mentioned above, the origin of this magnetic behavior could be due to the fact that the interhelicoidal M-M distance is fairly small and, therefore, the interaction between metal ions of adjacent molecules takes place [21,32,34].

Thus, taking into account the above background, the magnetic properties of the crystalline samples of both copper(II) [Cu_2_(L^1^)_2_]·4CH_3_CN and [Cu_2_(L^2^)_2_]·CH_3_CN helicates were studied by DC magnetic susceptibility and EPR spectroscopy.

**Figure 8:**
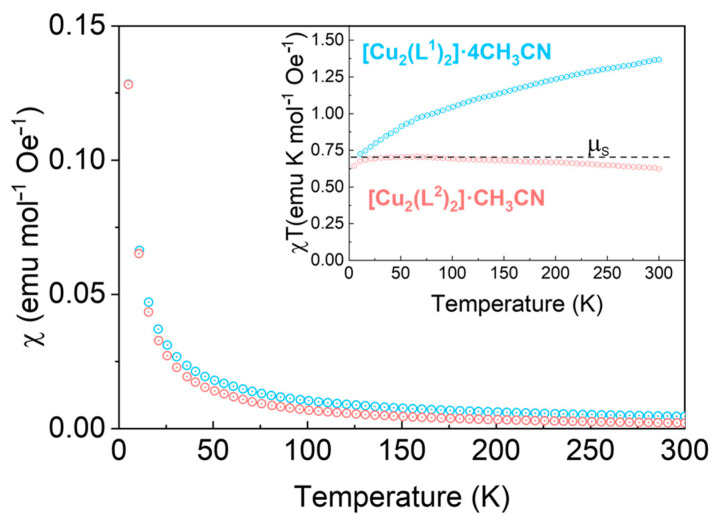
Temperature dependence of the molar magnetic susceptibility, χ, for [Cu_2_(L^1^)_2_]·4CH_3_CN and [Cu_2_(L^2^)_2_]·CH_3_CN helicates, measured at H = 100 Oe. The inset shows the temperature dependence of χT, proportional to the magnetic moment of the samples.

The temperature dependence of the magnetic susceptibility, χ, is shown in Figure 8. At first sight both compounds show a Curie-like behavior, without any hint of magnetic ordering down to 5 K. The two copper complexes show a χ_M_T ≈ 0.7 emu K mol^−1^ at low temperature, close to the χ_M_T ≈ 0.75 emu K mol^−1^ expected for a molecule with two independent Cu^2+^ ions with spin-only contribution (μ = 1.73 μ_B_). However, increasing temperature enhances χ_M_T for the [Cu_2_(L^1^)_2_]·4CH_3_CN helicate. This behavior is similar to that previously observed by us for a network assembled from Cu(II) helicates [21]. On the other hand, χ_M_T decreases slightly when increasing temperature in [Cu_2_(L^2^)_2_]·CH_3_CN.

Considering the total orbital contribution to the magnetic moment in Cu^2+^ ions will result in a μ = 3.54 μ_B/Cu_, and hence χ_M_T ≈ 3.5 emu K mol^−1^ for a lattice with two Cu sites. In the tetrahedral d^9^ configuration, the unpaired electron can occupy the dxz or dyz orbitals, so that the complex acquires an orbital angular momentum. The observed increase in χ_M_T in [Cu_2_(L^1^)_2_]·4CH_3_CN suggests a substantial orbital contribution from a partially distorted octahedral configuration (attributed to acetonitrile coordination), whose orbital occupation changes with temperature. 

**Figure 9:**
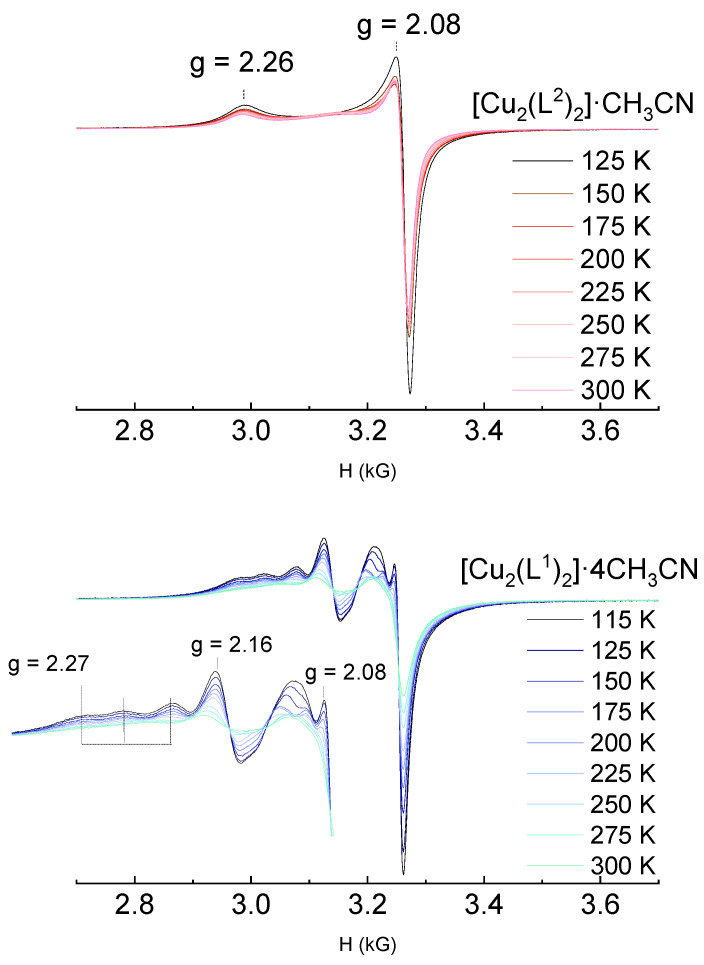
EPR spectra of [Cu_2_(L^2^)_2_]·CH_3_CN (**top**) and [Cu_2_(L^1^)_2_]·4CH_3_CN (**bottom**) helicates at different temperatures. The inset shows an enlarged portion of the spectrum.

The differences in the local coordination of copper in both complexes is further demonstrated by the differences observed in the EPR spectra of Cu^2+^ species, shown in Figure 9. The [Cu_2_(L^2^)_2_]·CH_3_CN shows the typical EPR spectrum for an axial complex of Cu^2+^ (S = 1/2) with g//(≈2.26) > gx⊥(≈2.08). These values are in the range reported for copper tetracoordinated by two oxygen and two nitrogen atoms [N_2_O_2_] [35,36]. The hyperfine coupling with the copper nucleus (I = 3/2) is not resolved at g//, which could be due to broadening by dipole–dipole interactions.

On the other hand, [Cu_2_(L^1^)_2_]·4CH_3_CN shows a more complex spectrum, consistent with a distorted structure, which could justify a larger and temperature-dependent orbital contribution to χ_M_T, discussed before. The contribution from two copper sites cannot be discarded, and a complete elucidation of the EPR spectrum of this helicate requires further investigation.

The differences observed in the magnetic behavior of the two reported helicates show that the position of the tert-butyl group in para (H_2_L^1^ ligand) or ortho (H_2_L^2^ ligand) with respect to the phenol group affects to the magnetic behavior of the compounds and, therefore, that the magnetic properties in the helicates can be modulated by small structural changes in the ligands.

## 3. Materials and Methods

All solvents, 4,4′-methylenedianiline, 3-tert-butyl-2-hydroxybenzaldehyde, 5-tert-butyl-2-hydroxybenzaldehyde and copper plates, were purchased from commercial sources and were used without purification. Melting points were determined using a BUCHI 560 instrument. Elemental analysis of compounds (C, N and H) was carried out on a FISONS EA model 1108 analyzer. Infrared spectra were recorded from 4000 to 500 cm^−1^ on a BRUKER FT-MIR spectrophotometer model VERTEX 70V in solid state using KBr pellets. Mass spectra were obtained using Bruker Microtof spectrometers for the ESI+ technique (electrospray ionization in positive mode) and Bruker Autoflex for the MALDI technique (matrix assisted laser desorption/ionization), both coupled to a time-of-flight (TOF) analyzer. A Varian Inova 400 spectrometer was employed to record the ^1^H NMR spectra operating at room temperature using acetone-d_6_ as the deuterated solvent. Chemical shifts are reported as *δ* (in ppm). 

### 3.1. Synthesis and Characterization of the Schiff Base Ligands H_2_L^1^ and H_2_L^2^

H_2_L^1^: 0.5 g (2.5 mmol) of 4,4′-methylenedianiline and 0.88 mL (5.0 mmol) of 5-tert-butyl-2-hydroxybenzaldehyde were dissolved in absolute ethanol (50 mL), adding a catalytic amount of p-toluensulfonic acid. The reaction mixture was heated under reflux with magnetic stirring for 4 h, using a Dean–Stark manifold to remove water and promote ligand formation. The resulting solution was concentrated to half volume and cooled, resulting in the formation of an orange precipitate. This solid was filtered and air-dried. Yield: 0.914 g (70%); m.p.: 155–160 °C; elemental analysis: % theoretical (C_35_H_38_N_2_O_2_) C, 81.0; N, 5.4; H, 7.4; experimental C, 81.4; N, 5.4; H, 7.3; IR (cm^−1^) ν: 3443 w (O-H); 2957 s (C-H); 1622 s (C=N); 1265 m (C-O); 820 vs. (CH_2_); ESI+ (*m*/*z*): 519.30 [H_2_L^1^ + H]^+^; ^1^H-NMR (400 MHz, acetone-d_6_, δ (m, nH, Hx, J)): 12.98 (s, 2H, H_1_), 8.92 (s, 2H, H_2_), 7.91 (d, J = 2.5 Hz, 2H, H_3_); 7.47 (dd, J = 8.7, 2.5 Hz, 2H, H_4_), 7.37 (s, 8H, H_5_ + H_6_); 6.89 (d, J = 8.7 Hz, 2H, H_7_); 4.08 (s, 2H, H_8_), 1.32 (s, 18H, H_9_).

H_2_L^2^: 0.5 g (2.5 mmol) of 4,4′-methylenedianiline and 0.88 mL (5.0 mmol) of 3-tert-butyl-2-hydroxybenzaldehyde were dissolved in absolute ethanol (50 mL). Then, a catalytic amount of p-toluensulfonic acid was added and the reaction mixture was heated under reflux with magnetic stirring for 4 h, using a Dean–Stark manifold to remove water and promote ligand formation. The resulting solution was concentrated to half volume and cooled, resulting in the formation of a yellow precipitate. This solid was filtered and dried in air. Yield: 1.02 g (78%); m.p.: 105–110 °C; elemental analysis: % theoretical (C_35_H_38_N_2_O_2_) C, 81.0; N, 5.4; H, 7.4; experimental C, 80.8; N, 5.4; H, 7.2; IR (cm^−1^) ν: 3437 vw (O-H); 2955 s (C-H); 1616 vs. (C=N); 1277 m (C-O); 745 s (CH_2_); ESI+ (*m*/*z*): 519.30 [H_2_L^2^ + H]^+^; ^1^H-NMR (400 MHz, acetone-d_6_, δ (m, nH, Hx, J)): 14.01 (s, 2H, H_1_), 8.90 (s, 2H, H_2_), 7.44–7.37 (m, 12H, H_3_-H_6_); 6.89 (t, J = 7 Hz, 2H, H_7_); 4.09 (s, 2H, H_8_), 1.44 (s, 18H, H_9_).

### 3.2. Synthesis and Characterization of the Neutral Copper(II) Dihelicates

The neutral copper(II) helicates were obtained by electrochemical synthesis using acetonitrile as solvent, applying a current intensity of 10 mA and potential values in the interval of 10–15 V. As an example, we describe below the electrochemical synthesis of the [Cu_2_(L^1^)_2_] helicate.

The electrochemical cell can be denoted as Pt(-)|H_2_L^1^+ CH_3_CN|Cu(+). The H_2_L^1^ ligand (0.05 g, 0.10 mmol) was previously dissolved in acetonitrile (80 mL) and a small amount of tetraethylammonium perchlorate was added to act as a conducting electrolyte. The electrolytic reaction was carried out under N_2_(g) atmosphere at 10 mA and 13.0 V for 31 min. The resulting solution was concentrated, giving rise to a brown solid that was filtered off and dried in vacuo. Caution! Although perchlorate salts were used in very small quantities in these reactions, they are potentially explosive and should be used with care.

The main analytical and characterization data of both copper(II) complexes are given below.

[Cu_2_(L^1^)_2_]: Brown solid. Yield: 0.068 g (61%); m.p.: >300 °C; Ef = 0.6 mol·F^−1^; elemental analysis: % theoretical (C_70_H_72_N_4_O_4_Cu_2_) C, 72.5; N, 4.8; H, 6.2; experimental C, 70.9; N, 4.8; H, 6.2; IR (cm^−1^) ν: 3397 br (O-H); 2954 m (C-H); 1618 vs. (C=N); 1255 m (C-O); 833 s (CH_2_); MALDI-TOF (m/z): 1159.42 [Cu_2_(L^1^)_2_ + H]^+^. By slow evaporation of the mother liquor from the synthesis, brown crystals suitable for X-ray diffraction studies were obtained ([Cu_2_(L^1^)_2_]·4CH_3_CN).

[Cu_2_(L^2^)_2_]·CH_3_CN: Brown solid. Yield: 0.079 g (68%); m.p.: >300 °C; Ef = 0.5 mol·F^−1^; elemental analysis: % theoretical (C_72_H_75_N_5_O_4_Cu_2_) C, 72.0; N, 5.8; H, 6.3; experimental C, 71.5; N, 5.3; H, 6.2; IR (cm^−1^) ν: 2953 m (C-H); 1611 s (C=N); 1246 w (C-O); 750 s (CH_2_); MALDI-TOF (*m*/*z*): 1159.42 [Cu_2_(L^2^)_2_ + H]^+^. By slow evaporation of the mother liquor from the synthesis, brown crystals suitable for X-ray diffraction studies were obtained ([Cu_2_(L^2^)_2_]·CH_3_CN).

### 3.3. X-ray Crystallography

Crystallographic data for both copper(II) dihelicates ([Cu_2_(L^1^)_2_]·4CH_3_CN) and ([Cu_2_(L^2^)_2_]·CH_3_CN) were collected at 100 K on a Bruker D8 VENTURE diffractometer equipped with a CCD detector, using an MoK (α) graphite monochromator (λ = 0.71073 Å). The data were treated with APPEX3v2018.7-2 software for both compounds.

In all cases, an absorption correction (SADABS) [37] was applied to the measured reflections. Structures were solved with SHELXT2018/2 [38]. All structures were refined using SHELXL2018/3 [39]. The hydrogen atoms were included in the model in geometrically calculated and refined positions. The images included in this chapter were prepared using Mercury [39]. CCDC no. 2257783 and 2257784 contain the supplementary crystallographic data for the [Cu_2_(L^1^)_2_]·4CH_3_CN and [Cu_2_(L^1^)_2_]·CH_3_CN dihelicates.

### 3.4. EPR Spectroscopy

EPR spectra of both copper(II) helicates [Cu_2_(L^1^)_2_]·4CH_3_CN and [Cu_2_(L^2^)_2_]·CH_3_CN were recorded at different temperatures using a Bruker EMX spectrometer operating at 9.5 GHz (X-Band).

### 3.5. Magnetic Susceptibility Measurements

DC magnetic susceptibility measurements for microcrystalline copper(II) helicates were performed at different fields in an MPMS SQUID magnetometer from Quantum Design, from 5–300 K. 

## 4. Conclusions

Two novel Cu(II) neutral dinuclear helicates were isolated using an electrochemical methodology and precursor Schiff base ligands functionalized with bulky tert-butyl groups in ortho and para positions. The discrete crystal structures of both copper(II) compounds [Cu_2_(L^1^)_2_]·4CH_3_CN and [Cu_2_(L^1^)_2_]·CH_3_CN confirm their helicoidal dinuclear nature. These structures are extended through the establishment of weak π-π or CH···π stacking interactions, with the intermolecular metal distance being smaller than the distance between the metal ions within the molecule, especially in the case of [Cu_2_(L^1^)_2_]·4CH_3_CN with the external tert-butyl groups located far away from the binding domains, thus confirming the influence of the bulky group location. This structural fact also influences the magnetic properties of the helicates in terms of local environments of the Cu(II) ions, but this finding will require further studies.

## Data Availability

Crystallographic data for [Cu_2_(L^1^)_2_]·4CH_3_CN and [Cu_2_(L^2^)_2_]·CH_3_CN were deposited into the Cambridge Crystallographic Data Centre, CCDC 2257783 and 2257784. These data can be obtained free of charge via www.ccdc.cam.ac.uk/data_request/cif, by emailing data_request@ccdc.cam.ac.uk, or by contacting The Cambridge Crystallographic Data Centre, 12 Union Road, Cambridge CB2 1EZ, UK; Fax: +44-1223-336033.

## Supplementary Materials

The supporting information can be downloaded at: https://www.mdpi.com/article/10.3390/ijms24108654/s1.
