# Supplementary material for: Schiff Bases Functionalized with T-Butyl Groups as Adequate Ligands to Extended Assembly of Cu(II) Helicates
Source: Int J Mol Sci. 2023 May 12;24(10):8654. doi: 10.3390/ijms24108654 (PMC10218674; doi:10.3390/ijms24108654)
Supplement: Supplementary file 1 [file ijms-24-08654-s001.zip › ijms-2393818-supplementary.pdf]

International Journal of *Molecular Sciences*

MDPI

Supporting Information

Schiff bases functionalized with t-butyl groups as adequate ligands to extended assembly of Cu(II) helicates

Sandra Fernández-Fariña,[1,*] Isabel Velo-Heleno,[1] Miguel Martínez-Calvo,[1] Marcelino Maneiro,[2] Rosa Pedrido[1,*] and Ana M. González-Noya[1]

1 Departamento de Química Inorgánica, Facultade de Química, Campus Vida, Universidade de Santiago de Compostela, 15782 Santiago de Compostela, Spain

2 Departamento de Química Inorgánica, Facultade de Ciencias, Universidade de Santiago de Compostela, 27002 Lugo, Spain

* Correspondence: sandra.fernandez.farina@usc.es (S.F.-F.); rosa.pedrido@usc.es (R.P.)

1. Schiff base ligands H_2L^1 and H_2L^2

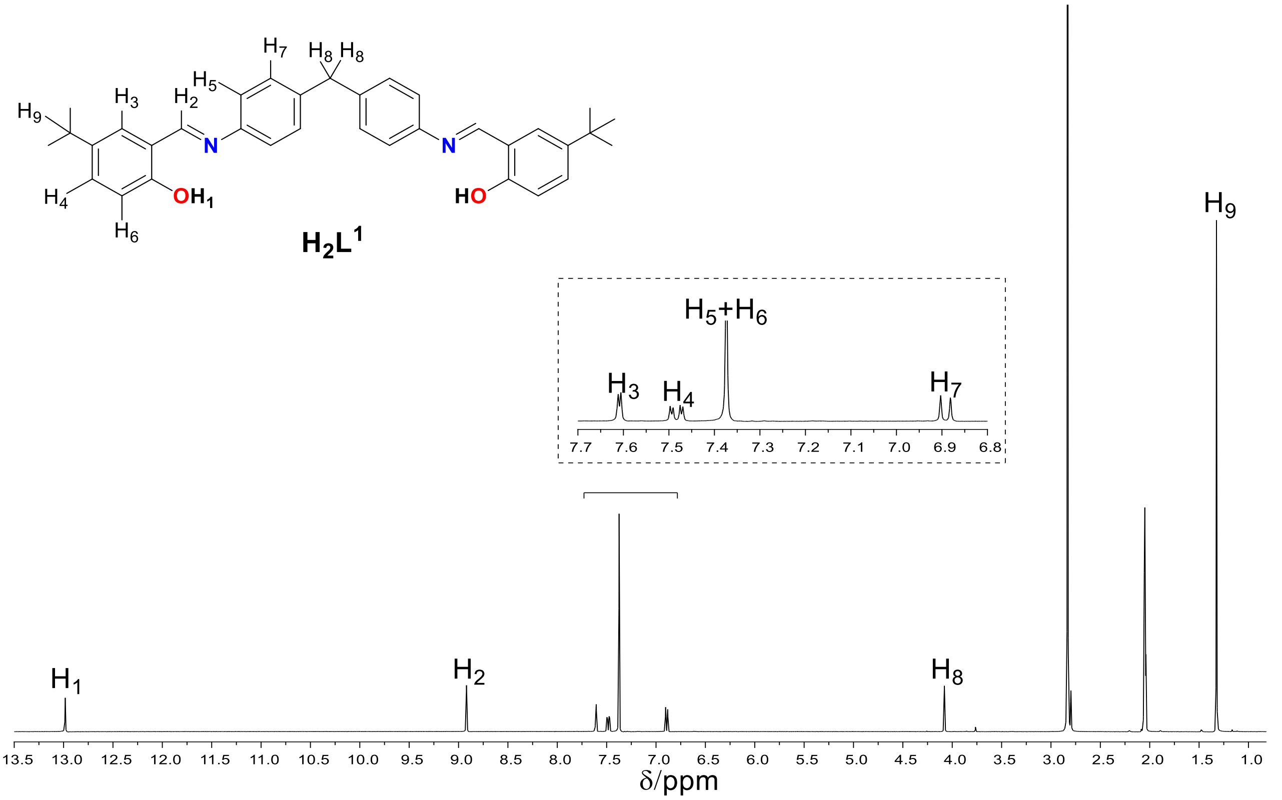

Figure S1. ^{1}H NMR spectra of H_2L^1 (400 MHz, acetone-d_6, r.t., δ (m, nH, Hx, J)): 12.98 (s, 2H, H_1), 8.92 (s, 2H, H_2), 7.91 (d, J= 2.5 Hz, 2H, H_3); 7.47 (dd, J= 8.7, 2.5 Hz, 2H, H_4), 7.37 (s, 8H, H_5+H_6); 6.89 (d, J= 8.7 Hz, 2H, H_7); 4.08 (s, 2H, H_8), 1.32 (s, 18H, H_9).

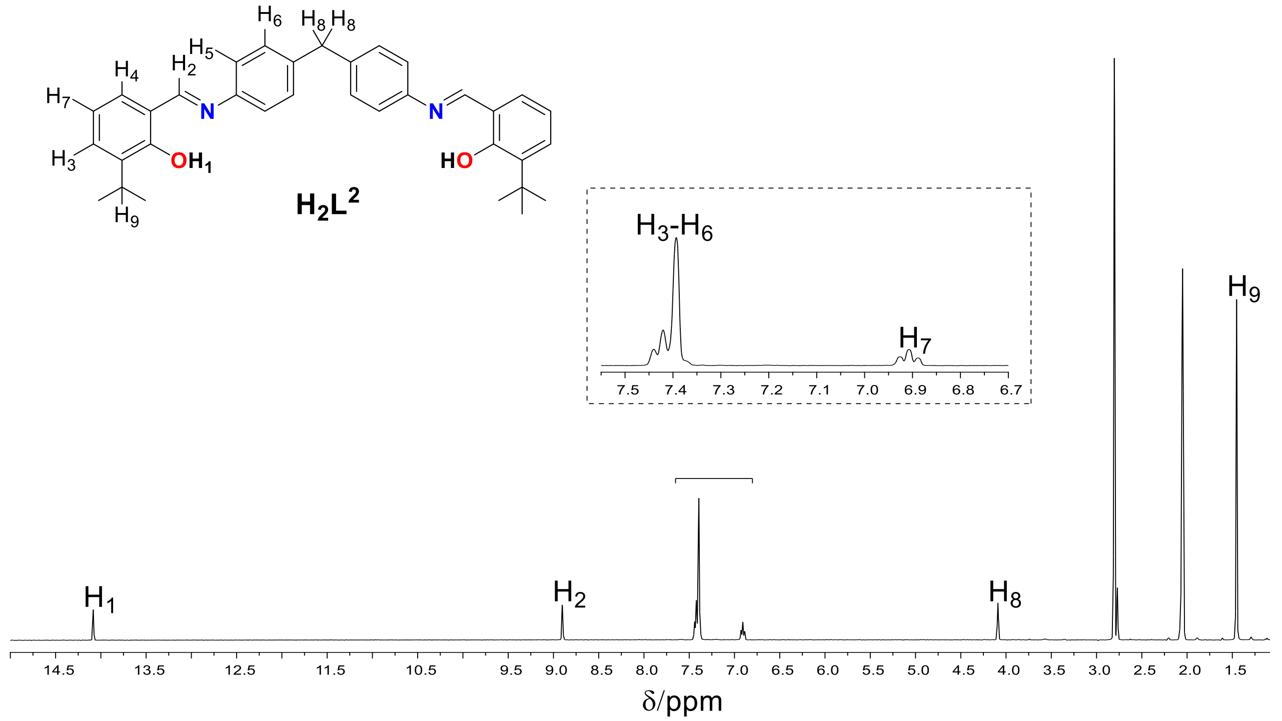

Figure S2. ^{1}H NMR spectra of H_2L^2 (400 MHz, acetone-d_6, r.t., δ (m, nH, Hx, J)): 14.01 (s, 2H, H_1), 8.90 (s, 2H, H_2), 7.44-7.37 (m, 12H, H_3-H_6); 6.89 (t, J= 7 Hz, 2H, H_7); 4.09 (s, 2H, H_8), 1.44 (s, 18H, H_9).

2. Copper(II) helicates derived from Schiff base ligands

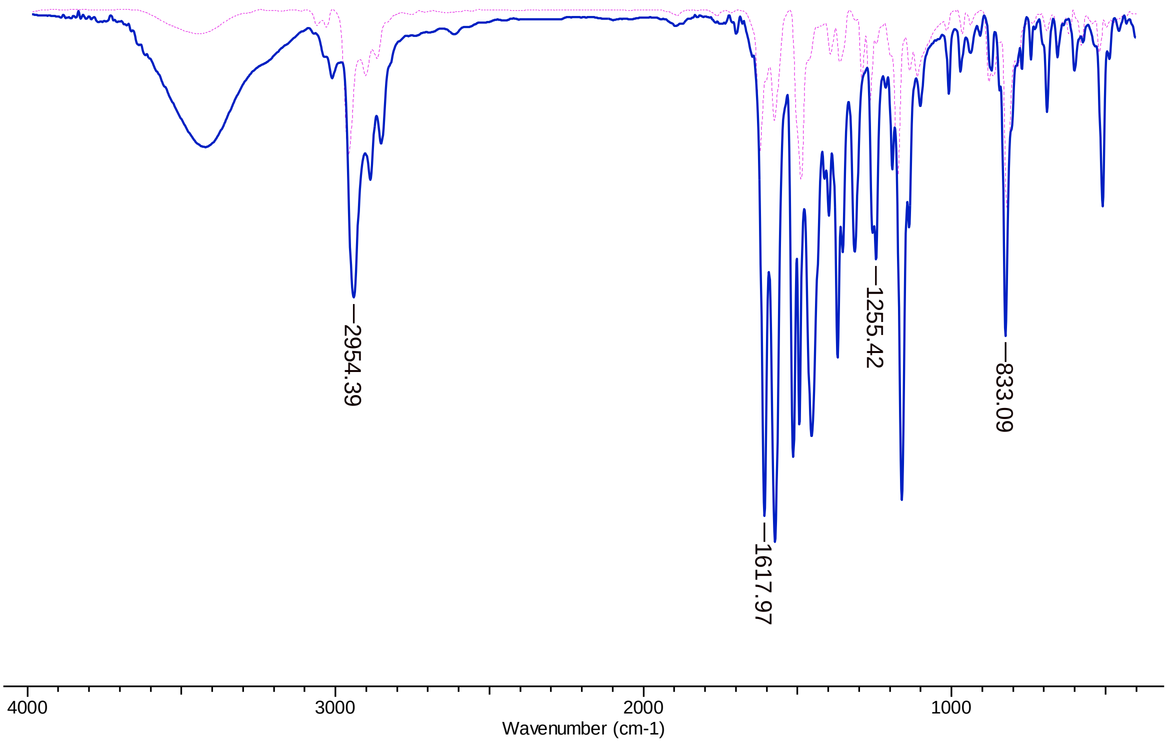

Figure S3. Infrared spectra superposition of H_2L^1 (pink) and $[Cu_2(L^1)_2]$ (blue).

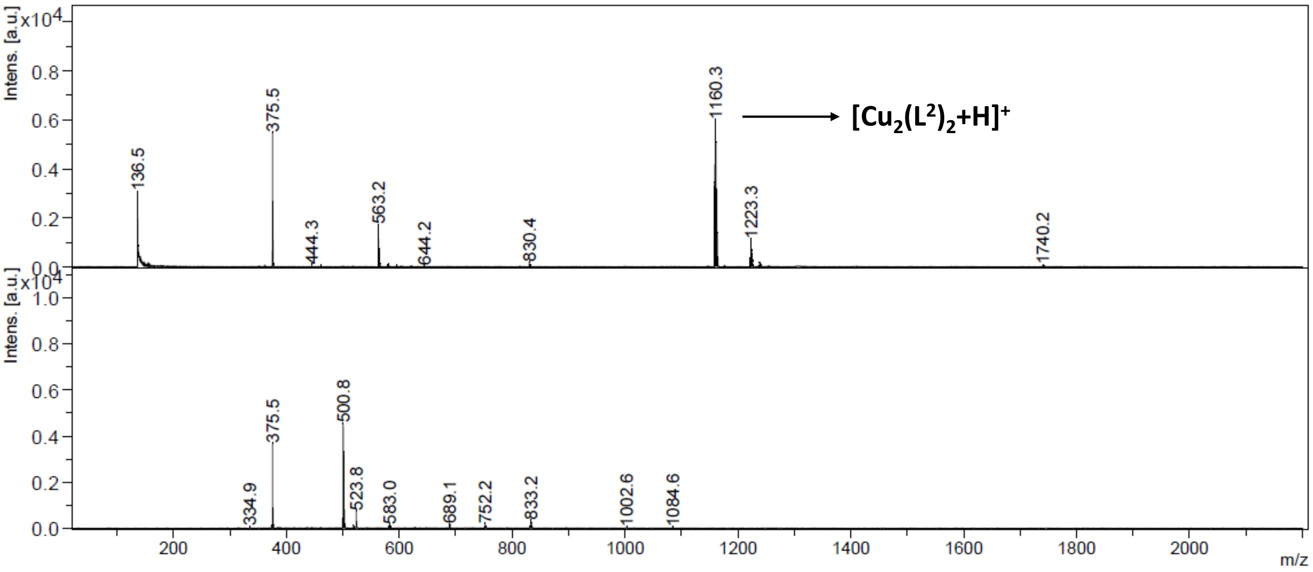

Figure S4. Mass spectra of $[Cu_2(L^2)_2]{\cdot}CH_3CN$.

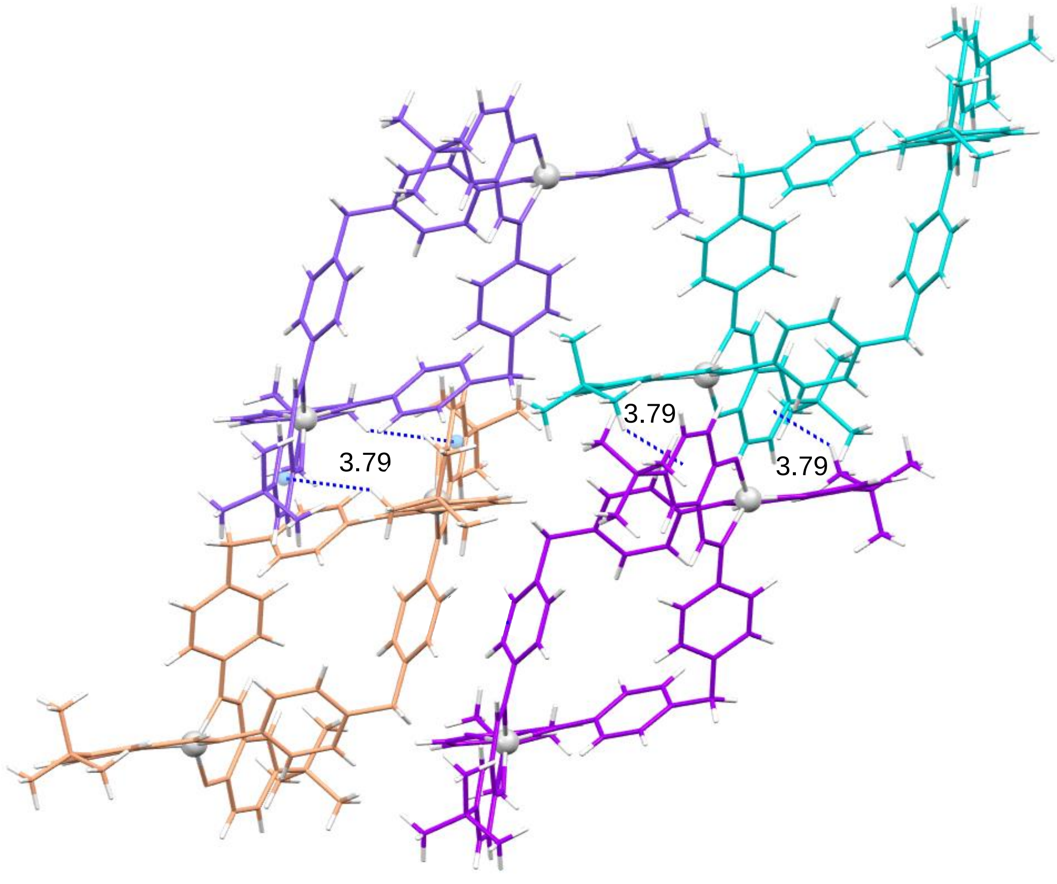

Figure S5. Intermolecular CH···π interactions in the crystal lattice of the $[Cu_2(L^1)_2]·4CH_3CN$ helicate.

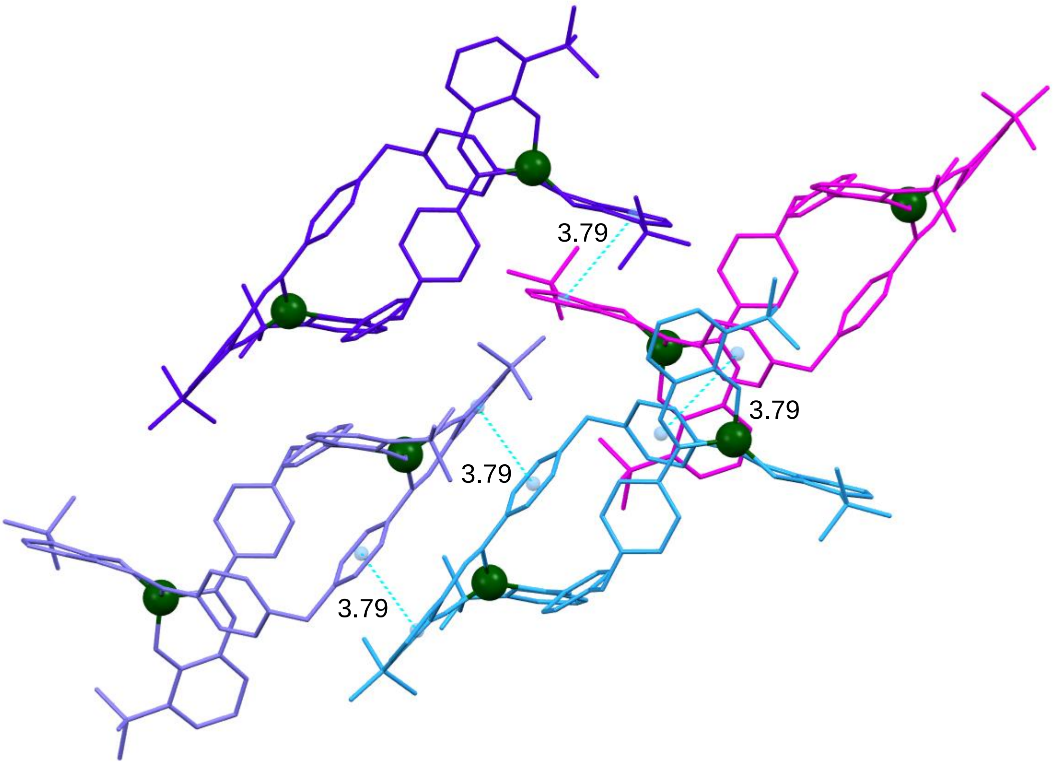

Figure S6. Intermolecular π ···π interactions in the crystal lattice of the $[Cu_2(L^2)_2]·CH_3CN$ helicate.

Table S1. Main crystallographic data for helicates $[Cu_2(L^1)_2]\cdot 4CH_3CN$ and $[Cu_2(L^2)_2]\cdot CH_3CN$.

	$[Cu_2(L^1)_2]\cdot 4CH_3CN$	$[Cu_2(L^2)_2]\cdot CH_3CN$
Molecular formula	$C_{72}H_{75}N_5O_4Cu_2$	$C_{72}H_{75}N_5O_4Cu_2$
Molecular weight	1329.11	1201.45
Spatial group	*I*2/*a*	$P\bar{1}$
Crystalline system	Monoclinic	Triclinic
Crystal size/mm	0.20 × 0.19 × 0.07	0.05 × 0.04 × 0.02
a/Å	18.7980(5)	11.4703(5)
b/Å	17.0029(5)	13.3929(5)
c/Å	23.3318(9)	20.4608(7)
α/º	90	95.462(3)
β/º	109.65 (10)	101.556(3)
γ/º	90	100.206(3)
Temperature/K	100	100
Volume/Å^3	7023.0 (4)	2667.0(2)
Z	4	2
Measured reflexions	45292	41389
Unique reflexions [R_{int}]	7172 [0.044]	10230 [0.159]
μ/mm^{-1}	0.661	1.300
Residues/e Å^{-3}	2.11 and -0.53	0.35 and -0.79
R	0.065	0.068
*w*R	0.109	0.187

Table S2. Main bond distances and angles for $[Cu_2(L^1)_2]\cdot 4CH_3CN$ helicate.

Bond distances (Å)					
N1—Cu1	1.967 (2)	O1—Cu1	1.8935 (18)	N1—Cu1	1.967 (2)
N2—Cu1i	1.962 (2)	O2—Cu1i	1.8907(17)	N2—Cu1i	1.962 (2)
**Bond angles (⁰)**					
O1—Cu1—O2^i	92.07(8)	O2—Cu1i—N1^i	142.00(9)	O1—Cu1—O2^i	92.07(8)
O2i—Cu1—N2^i	94.90(8)	O1i—Cu1i—N1^i	94.89(8)	O2i—Cu1—N2^i	94.90(8)
O1—Cu1—N2^i	144.84(9)	N2—Cu1i—N1^i	100.41(8)	O1—Cu1—N2^i	144.84(9)

Table S3. Main bond distances and angles for $[Cu_2(L^2)_2]\cdot CH_3CN$ helicate.

Bond distances (Å)					
N1—Cu1	1.963 (5)	O1—Cu1	1.885 (4)	N1—Cu1	1.963 (5)
N2—Cu2	1.943 (4)	O2—Cu2	1.903 (4)	N2—Cu2	1.943 (4)
N3—Cu2	1.973 (5)	O3—Cu2	1.875 (4)	N3—Cu2	1.973 (5)
N4—Cu1	1.942 (5)	O4—Cu1	1.895 (3)	N4—Cu1	1.942 (5)
**Bond angles (⁰)**					
O4—Cu1—O1	95.32 (16)	O2—Cu2—O3	92.28 (16)	O4—Cu1—O1	95.32 (16)
O4—Cu1—N1	139.98 (18)	O2—Cu2—N3	147.87 (18)	O4—Cu1—N1	139.98 (18)
O1—Cu1—N1	93.02 (18)	O3—Cu2—N3	94.45 (18)	O1—Cu1—N1	93.02 (18)
O4—Cu1—N4	94.07 (17)	O2—Cu2—N2	92.71 (18)	O4—Cu1—N4	94.07 (17)
O1—Cu1—N4	146.43 (19)	O3—Cu2—N2	150.31 (19)	O1—Cu1—N4	146.43 (19)
N1—Cu1—N4	100.18 (19)	N3—Cu2—N2	96.72 (19)	N1—Cu1—N4	100.18 (19)
O4—Cu1—O1	95.32 (16)	O2—Cu2—O3	92.28 (16)	O4—Cu1—O1	95.32 (16)
O4—Cu1—O1	95.32 (16)	O2—Cu2—O3	92.28 (16)	O4—Cu1—O1	95.32 (16)

Table S4. Main H-bond distances and angles for $[Cu_2(L^1)_2]\cdot 4CH_3CN$ helicate.

D—H···*A*	*D*—H	H···*A*	*D*···*A*	*D*—H···*A*
C7—H7···N4	0.95	2.67	3.47	142
C19—H19···N3	0.95	2.60	3.54	169
C39—H39···O2	0.98	2.66	3.14	111

Table S5. Main H-bond distances and angles for $[Cu_2(L^2)_2]\cdot CH_3CN$ helicate.

D—H···*A*	*D*—H	H···*A*	*D*···*A*	*D*—H···*A*
C19—H19···N5	0.95	2.48	3.41	169
